# LINC00941 is a diagnostic biomarker for lung adenocarcinoma and promotes tumorigenesis through cell autophagy

**DOI:** 10.1111/jcmm.70076

**Published:** 2024-10-11

**Authors:** Qin Yang, Xi Yong, Xiaoli Chen, Rong Huang, Xiaolin Wang, Zhengmin Xu, Wei Chen

**Affiliations:** ^1^ School of Basic Medical Sciences Chengdu University of Traditional Chinese Medicine Chengdu China; ^2^ Innovative Institute of Chinese Medicine and Pharmacy Chengdu University of Traditional Chinese Medicine Chengdu China; ^3^ Department of Vascular Surgery Affiliated Hospital of North Sichuan Medical College Nanchong China; ^4^ Department of Pathology, Basic Medicine and Forensic Medicine College North Sichuan Medical College Nanchong China; ^5^ School of Pharmacy, Institute of Materia Medical North Sichuan Medical college Nanchong China; ^6^ Traditional Chinese Medicine for Prevention and Treatment of Musculoskeletal Diseases Key Laboratory of Nanchong City Nanchong China

**Keywords:** autophagy, LINC00941, lung adenocarcinoma, PI3K/AKT/mTOR pathway

## Abstract

Non‐small cell lung cancer (NSCLC) is a lethal malignancy. There is mounting evidence indicating that lncRNAs are crucial players with dual roles as both biomarkers and regulators across various cancers. It was reported that LINC00941 plays a cancer‐promoting role in NSCLC. However, its impact on tumour autophagy remains poorly understood. In this study, we developed a risk assessment model and identified an autophagy‐related lncRNA LINC00941, which has independent predictive and early diagnostic potential. Using RT‐qPCR analysis, we confirmed the upregulation of LINC00941 in tumour tissues and cell lines of human lung adenocarcinoma (LUAD). Functional assays, such as CCK8, colony formation and xenograft models, demonstrated the cancer‐promoting activity of LINC00941 both in vitro and in vivo. Further analysis using Western blotting analysis, mRFP‐GFP‐LC3 double fluorescence lentivirus vector and transmission electron microscopy (TEM) confirmed that the knockdown of LINC00941 triggered autophagy. These results indicate that knockdown of LINC00941 induces autophagy and impairs the proliferation of LUAD. Therefore, we propose LINC00941 as an independent biomarker for early diagnosis as well as a therapeutic target in LUAD.

## 1 INTRODUCTION

Non‐small cell lung cancer (NSCLC) is a prevalent malignancy, with lung adenocarcinoma (LUAD) as the predominant pathological subtype, accounting for 78% of cases.^1^ While advancements in tumour treatments, such as targeted therapy and immunotherapy, have improved overall care, the survival rate for LUAD remains low, with only 10%–20% of patients surviving 5 years after diagnosis.^2^ This is primarily attributed to the absence of reliable early diagnostic and therapeutic indicators.^2^, ^3^, ^4^ Therefore, it is urgently needed to identify specific diagnostic biomarkers and therapeutic targets.^5^, ^6^

Low‐dose CT is recommended as an early screening method that could reduce mortality by 20.0%, but it also had a false positive rate of 23.3%.^7^ The low‐dose CT combination with cancer markers is commonly performed in clinical. However, previous studies have shown that the sensitivity of tumour markers (CEA, NES, SCC and CYFRA21‐1) recommended for lung cancer does not exceed 25%, or even less than 10%.^8^, ^9^ On the contrary, the dysregulated expression of long non‐coding RNAs (lncRNAs), detectable in blood of patients, has been reported to play a role in various aspects of tumorigenesis, progress and treatment, making them potential indicators of diagnosis and treatment.^10^, ^11^, ^12^, ^13^ Additionally, numerous studies on cancers have shown that lncRNAs are involved in tumour development by influencing cell autophagy. For instance, the knockdown of lncRNA RP11‐476D10.1 can inhibit papillary thyroid carcinoma cell proliferation by activating autophagy.^14^ Consequently, the identification of lncRNAs closely correlated with autophagy assumes paramount importance in understanding their diagnostic and therapeutic roles in tumours.

The impact of autophagy on cancer is dual and complex. Generally, autophagy tends to be the way that cells maintain survival.^15^, ^16^, ^17^ However, under certain adverse conditions, it can also induce cell death, manifesting anti‐cancer effects.^18^, ^19^ Besides, the PI3K/AKT/mTOR pathway plays vital roles in cellular autophagy and proliferation in various cancers.^20^, ^21^ Abnormal activation of AKT and mTOR have been found in most LUAD.^22^ The mTOR positively regulates protein synthesis such as cell proliferation, while negatively regulates catabolic processes such as autophagy.^23^, ^24^, ^25^ This underscores the significance of the PI3K/AKT/mTOR pathway in maintaining the delicate balance between proliferation and autophagy. LINC00941, also known as lncRNA‐MUF, functions as a cancer‐associated mesenchymal stem cell upregulated factor.^26^ Previous studies have highlighted the intricate regulatory mechanisms of LINC00941, demonstrating its influence at both transcriptional and post‐transcriptional levels, thereby impacting tumour proliferation, migration and invasion.^27^ It was reported that LINC00941 was demonstrated to promote the development of NSCLC by up‐regulating VEGFA and promoting angiogenesis.^28^ However, few studies have been explored the underlying regulatory mechanism of LINC00941 on LUAD cell autophagy.

In this study, we identified lncRNA LINC00941 as an early independent prognostic indicator for patients diagnosed with LUAD through bioinformatic methods. Furthermore, we validated the level of LINC00941 in 67 pairs of LUAD and adjacent normal tissues. Our investigation involved exploring its effects on tumour cell proliferation and autophagy, as well as deciphering potential regulatory mechanisms. Finally, we found that LINC00941 exerts inhibitory effects on cellular autophagy and facilitates the proliferation of LUAD cells.

## 2 MATERIALS AND METHODS

### 2.1 Exploring autophagy‐associated lncRNAs

Data on the expression levels of RNA fragments per kilobase million (FPKM) and corresponding clinical profiles for a total of 522 cases were acquired from TCGA‐LUAD dataset (https://portal.gdc.cancer.gov/), which has a large sample size and complete clinical information. A comprehensive collection of 222 genes associated with autophagy was acquired from Human Autophagy Database (HADb). The FPKM values of RNA‐Seq data were transformed into TPM (Transcripts Per Kilobase Million), and then the TPM values were normalized to log2 for further analysis. As previous studies have shown that the threshold for identifying autophagy‐associated lncRNAs through Pearson correlation analysis differs across various human cancers.^29^, ^30^ To ensure both the efficacy of the model and its suitability for clinical implementation, a threshold of |*R*| >0.5 and *p* < 0.001 was established based on our empirical analysis and previous literatures. Ultimately, a total of 1301 candidates were selected for further analysis.

### 2.2 Development and assessment of prognostic signature

The Cox regression model is commonly used for prognostic modelling. Univariate Cox regression analysis (survival R package) was conducted to filter out possible confounding factors and identify the lncRNAs significantly correlated with prognosis. The addition of L1 regularization in LASSO‐Cox regression analysis is conducive to screening out the lncRNAs that contribute the most to prognosis.^31^ Thus, the optimal gene combinations were explored by subjecting these candidates to LASSO‐Cox regression analysis (glmnet R package). The results of the LASSO‐Cox regression analysis were then incorporated into multivariate Cox regression analysis (survminer R package) for further identifying independent factors. The risk score for each individual was calculated by summing the regression coefficient of each lncRNA multiplied by its expression value. The principal component analysis (PCA) was performed to visualize the expression patterns in the high or low risk groups categorized by the median risk score. Additionally, the overall survival of the two risk groups was evaluated using Kaplan–Meier analysis. To visualize co‐expression relationships, the ggalluvial R package was employed. The independent predictive performance was evaluated using both univariate and multivariate Cox regression analyses. The area under the receiver operating characteristic curve (AUC) values were computed to verify the predictive accuracy of the risk score by using the timeROC R package.

### 2.3 Evaluation of the clinical value of LINC00941

Based on TCGA‐LUAD dataset, the Cox regression analysis (univariate and multivariate) and ROC curves (pROC R package) were used to assess the independent predictive and diagnostic performance of LINC00941 expression, respectively. The correlation between expression levels of LINC00941 and tumour size was assessed by Pearson correlation analysis based on 67 pairs of clinical samples. LINC00941 expression level and tumour size were derived from RT‐qPCR data and chest CT data, respectively.

### 2.4 Human LUAD tissues and cell lines

Initially, 39 pairs of LUAD specimens and their corresponding adjacent normal tissues were gathered for the preliminary screening of lncRNAs. Generally, the larger the sample size, the smaller the error. After identifying a potential biomarker, the sample size was expanded to 67 pairs to improve the accuracy and reliability of the conclusions, and the expression levels of this biomarker were subsequently examined in these clinical samples. All these samples of pathologically confirmed LUAD were obtained from the Affiliated Hospital of North Sichuan Medical College, which were approved by the Ethics Committee of this institute (No.2021ER053‐2). Meanwhile, we examined the relationship between LINC00941 levels in tumour tissues and clinicopathological features (Table 1). The A549, H1975, H1299 and HCC827 cell lines were from Procell Life (Wuhan, China). The BEAS‐2B and PC‐9 cell lines were kindly presented by professor Yin Chong (North Sichuan Medical College). The A549 and other cells were cultured in Ham's F‐12 K medium (Procell Life, China) and RPMI 1640 medium (Gibco, USA) supplemented with 10% FBS (Gibco, USA), respectively.

**TABLE 1:** Correlation of LINC00941 expression with clinicopathological factors in 67 LUAD patients.

Clinicopathological parameter	Number	LINC00941 expression		*p*‐Value
		High expression	Low expression	
Age (y)				
>65	28	13	15	0.5492
≤65	39	21	18	
Gender				
Female	36	15	21	0.1092
Male	31	19	12	
Smoking				
Yes	26	15	11	0.3651
No	41	19	22	
Anatomic location				
Left lung	36	19	17	0.7200
Right lung	31	15	16	
TNM stage				
I	57	26	31	0.0448*
II/III	10	8	2	
Tumour size (cm)				
≤1	7	1	6	0.0011*
1–2	29	10	19	
≥2	31	23	8	

*Note:* * Indicates statistical significant.

### 2.5 Real‐time quantitative PCR (RT‐qPCR)

RNA was extracted and reversed as previously reported.^32^ Subsequently, qPCR was performed using cDNA as template and β‐actin as an endogenous control. The primer sequences were listed in Table S1.

### 2.6 Cell transfection

Lentiviruses with shRNA (sh‐NC, sh‐LINC00941‐1, sh‐LINC00941‐2), or cDNA (oe‐NC, oe‐LINC00941) (Cyagen, Guangzhou, China) targeting LINC00941 were, respectively, used to infect H1299 or A549 and selected by puromycin to construct corresponding stable cell lines. In cell transfection, sh‐NC (non‐targeted sequence) was used as a silencing negative control, and oe‐NC (empty vector) was used as an overexpression negative control. The shRNA targeting sequences used in this study were as follows: sh‐LINC00941‐1, which was 5′‐ATGGACCAACTATGCTTATAACTCGAGTTATAAGCATAGTTGGTCCAT‐3′, and sh‐LINC00941‐2, which was 5′‐CAGCCAAATACTTTCAATAATCTCGAGATTATTGAAAGTATT‐3′. Both a negative control siRNA (si‐NC) and LINC00941‐specific siRNA (si‐LINC00941) were from Ribobio (Guangzhou, China). The siRNA targeting sequences were 5′‐GGCATACTGACAATACAAA‐3′.

### 2.7 RNA fluorescence in situ hybridization (FISH)

A549 and H1299 cells were seeded at a predetermined density to achieve a cell confluence of 60%–70% before the experiment. According to the RNA FISH kit instructions (Ribobio, Guangzhou, China), the cells were fixed, incubated with FISH probes (LINC00941, 18S, or U6), and subsequently subjected to DAPI staining, and detected by confocal microscope (Olympus, Japan). 18S was used as a marker for cytoplasm, U6 was used as a marker for nucleus.

### 2.8 Cell proliferation assays

A549 and H1299 with shRNA or cDNA targeting LINC00941 were seeded with 1500 cells in each well (96‐well plates) for the CCK8 assay. At specified time intervals (0 h, 24 h, 48 h, 72 h and 96 h), absorbance was measured at 450 nm by adding 10% CCK8 solution, subsequently generating the activity graphs. For the colony formation assay, 1000 cells of A549 and H1299 with shRNA or cDNA were cultured in 6‐well plates for a 14‐day period. Then the colonies were stained with 0.1% crystal violet and photographed.

### 2.9 Western blotting analysis

Protein extraction was conducted by employing RIPA buffer supplemented with a combination of a mixture of PMSF and phosphatase inhibitor. The cell lysate samples were subjected to separation via a 12% or 10% SDS‐PAGE, followed by transfer onto PVDF membranes (Merck‐Millipore, USA) through wet transfer technique. The membranes were incubated with the primary antibody that targets the protein of interest. The primary antibodies against seven antibodies were from CST: GAPDH (#97166), P62 (#8025) and LC3A/B (#12741). They were diluted at a ratio of 1:1000 to prepare working concentrations. Finally, antigen–antibody reactive bands were visualized with a chemiluminescence regent (Merk‐Millipore, USA). GAPDH was used as an internal control.

### 2.10 Transmission electron microscopy (TEM)

The cells were subjected to treatment with 1.5% glutaraldehyde for a minimum of 2 h at 4°C. Then, they were rinsed several times with PBS and fixed with l% osmium tetroxide in 0.1 mol/L PBS for 2 h at 25°C. Following another round of PBS washing, the cells were dehydrated with graded ethanol, immersed in epoxy resin overnight, embedded and polymerized at 60°C for 48 h. Finally, ultrathin sections were sliced and stained. The ultrastructure of cells was observed and captured under TEM (Hitachi, Japan).

### 2.11 Detection of autophagic flux

The mRFP‐GFP‐LC3 double fluorescence lentivirus (Genechem, Shanghai, China), containing HU6‐MCS‐Ubiqutin‐StubRFP‐SensGFP‐LC3‐IRES‐puromycin elements, were used to infect A549 and H1299 cells. These cells were subsequently subjected to select using puromycin. The stable cells were cultured on coverslips and reinfected with siRNA targeting LINC00941. The autophagic flux in cells was observed by confocal microscope (Olympus, Japan).

### 2.12 In vivo tumorigenicity assay

An in vivo tumorigenicity assay was conducted on 6‐week‐old male BALB/C nude mice that were divided into three groups using random number table (*n* = 5 per group). A subcutaneous injection of a 100 μl PBS suspension containing 5 × 10^6^ H1299 cells that had been undergone stable transfection with sh‐LINC00941‐1, sh‐LINC00941‐2, or sh‐NC was administered into the right armpit of each nude mouse. The weights of the mice and the volumes of their tumours were documented at 3‐day intervals over a period of 1 month until the mice were euthanized. All tumours were weighed and harvested for H&E staining and immunohistochemistry (IHC) analysis. Approval for the animal studies was granted by the Animal Research Ethics Committees at North Sichuan Medical College (No.202212).

### 2.13 IHC staining

After the deparaffinization and hydration process, tissue sections obtained from xenograft tumours in nude mice were immersed in a 3% H_2_O_2_ methanol solution to block endogenous peroxidase, boiled in a microwave oven for 20 min to repair antigen, incubated with anti‐Ki67 at 37°C for 30 min, stained with DAB, and then photographed.

### 2.14 Gene set enrichment analysis (GSEA)

The KEGG mainly includes GENES and PATHWAY databases, which help link genetic information with functional information.^33^ Hallmark gene sets are curated sets of genes that highlight specific biological processes or molecular pathways. These gene sets are designed to capture the essence of certain biological phenomena.^34^ GSEA is the preferred way for functional annotation.^35^ To investigate the regulatory mechanism of LINC00941 on LUAD, we performed GSEA on the genome‐wide expression matrix to explore the Hallmark and KEGG enrichment pathways. The FDR and NOM (nominal) *p*‐value was used to adjust for multiple hypothesis tests to control the proportion of false positives and estimate the significance level of the enrichment score, respectively. The signalling pathways with FDR <0.25 and NOM *p*‐value <0.05 were considered significant.

### 2.15 Statistical analysis

Data were conducted using R 4.0.3 and GraphPad Prism 6.0, and presented as mean ± SD. If data satisfied normality (Shapiro–Wilk normality test, *p* < 0.05), a two‐tailed Student's *t*‐test was used in comparisons between two groups; otherwise, Mann–Whitney U test were applied. For paired clinical data that did not satisfied normality, the Wilcoxon matched‐pairs signed rank test was applied to assess the statistical differences. Comparisons among multiple groups were performed by two‐tailed Student's *t*‐test or one‐way ANOVA followed by the Dunnett's or Tukey's post hoc test. *p* < 0.05 was considered statistically significant.

## 3 RESULTS

### 3.1 LncRNAs associate with autophagy and serve as prognostic signature of LUAD

**FIGURE 1:**
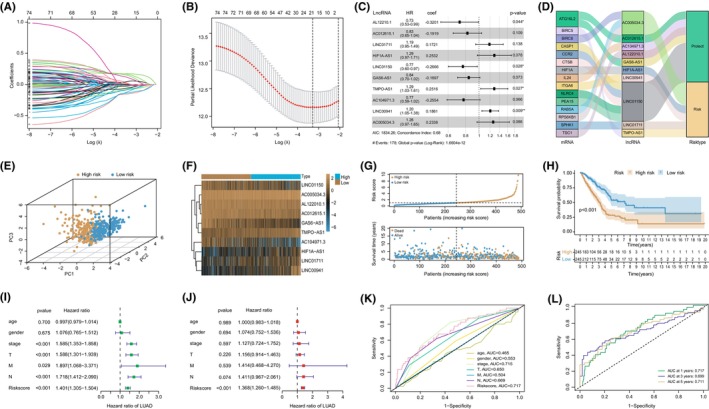
Establishment and evaluation of the risk signature with 10 autophagy‐related lncRNAs. (A, B) Log(λ) and the optimal log(λ) value of identified lncRNAs. (C) The 10 autophagy‐related lncRNAs were selected to establish risk signature by multivariate Cox regression analysis. (D) The visualization of the relationship between lncRNA and mRNA was achieved by a Sankey diagram. (E) The PCA shows the different distribution between the two risk groups. (F) The correlation between risk values and levels of lncRNA expression. (G) The scatter plots illustrate the survival status of patients with different risk values. (H) The Kaplan–Meier curves demonstrated superior survival outcomes in the low‐risk cohort. (I, J) The findings derived from both univariate and multivariate Cox regression analyses. (K) The AUC values for classifying low and high‐risk groups by using risk score and clinical factors. (L) The AUC values for predicting 1‐, 3‐ and 5‐year survival rates by using risk score.

To mining lncRNAs associated with autophagy, a total of 77 lncRNAs were acquired through univariate Cox regression analysis (Table S2). The determination of optimal combinations of lncRNAs for constructing the prognostic model was accomplished by employing LASSO‐Cox regression analysis (Figure 1A,B). As illustrated in Figure 1C, the risk signature was obtained by multivariate Cox regression analysis, including five risk‐factors lncRNAs (HIF1A‐AS1, TMPO‐AS1, AC005034.3, LINC00941 and LINC01711) with HR (hazard ratio) >1, and five protective‐factors lncRNAs (AL122010.1, LINC01150, AC104971.3, AC012615.1 and GAS6‐AS1) with HR <1. Further co‐expression analysis showed that these lncRNAs may target mRNAs related to inflammation, hypoxia, pyroptosis and autophagy, with LINC00941 showing a link to the autophagic gene ITGA6 (Figure 1D).

According to the PCA results, patients categorized as high or low risk, determined by the median score, exhibited different distribution patterns (Figure 1E). As shown in Figure 1F, the high‐risk groups exhibited elevated expression of the risk lncRNAs, whereas the low‐risk groups displayed an abundance of protective lncRNAs. Furthermore, the mortality rate of LUAD increased with a higher risk score, and the Kaplan–Meier plots illustrated that the groups with lower risk had a higher survival rate in contrast to the groups with higher risk (Figure 1G,H). The results of univariate Cox regression (HR: 1.401, *p* < 0.001) and multivariate Cox regression (HR: 1.368, *p* < 0.001) analyses indicated that the risk score was independent of other clinical factors (Figure 1I,J). The AUC value of the risk score for classifying low‐ and high‐risk groups was 0.717, surpassing the predictive capabilities of the other clinicopathological parameters in terms of patients' survival (Figure 1K). Additionally, the AUC values for predicting the survival rates were 0.717 (1 year), 0.699 (3 years) and 0.711 (5 years) (Figure 1L). These results demonstrated that the risk signature composed of 10 autophagy‐related lncRNAs was a significant and independent risk factor with a promising predictive performance, and the 10 lncRNAs could potentially serve as indicators for the diagnosis and prognosis of LUAD.

### 3.2 Validation of the 10 autophagy‐associated lncRNAs

**FIGURE 2:**
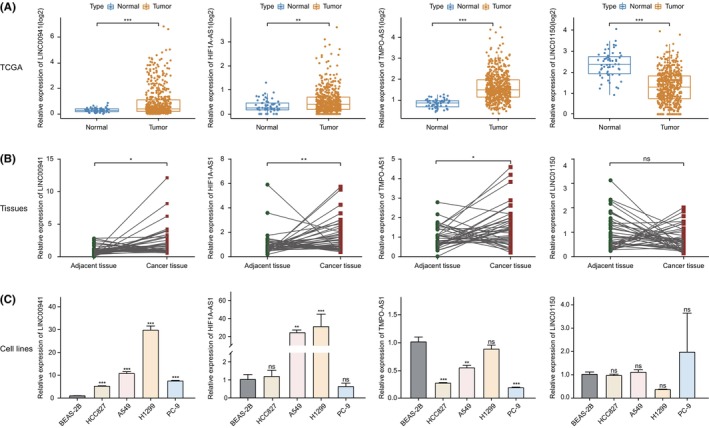
The expression level of the four autophagy‐related lncRNAs. (A) The expression levels of the four lncRNAs in normal and tumour samples from TCGA. Data were analysed by two‐tailed Student's *t*‐test or Mann–Whitney U test. (B) The expression levels of the four lncRNAs in 39 paired OF clinical samples was detected by RT‐qPCR. Data were analysed by Wilcoxon matched‐pairs signed rank test. (C) The expression levels of the four lncRNAs in cell lines (BEAS‐2B, HCC827, A549, H1299 and PC‐9). Data were analysed by one‐way ANOVA followed by the Dunnett's post hoc test and expressed as mean ± SD. *p < 0.05, **p < 0.01, ***p < 0.001.

To initially assess the expression levels of the 10 lncRNAs in clinical LUAD samples, a total of 39 sample pairs were included and evaluated using RT‐qPCR. The results of preliminary verification showed that the levels of three lncRNAs (LINC00941, HIF1A‐AS1 and TMPO‐AS1) between adjacent normal and tumour tissues were consistent with those observed in the TCGA database, while the levels of LINC01150 were not significantly different (Figure 2A,B). Moreover, in agreement with the results of tumour tissues, the upregulation of LINC00941 was also observed in four lung cancer cell lines (Figure 2C). In addition, six lncRNAs were excluded from subsequent experiments due to their lack of annotation in National Center for Biotechnology Information database, namely AC005034.3, LINC01711, AL122010.1, AC104971.3 and AC012615.1, or their absence of significant expression differences between adjacent and tumour tissues, such as GAS6‐AS1 (Figure S1). Therefore, based on these results, LINC00941 was chosen as a potential biomarker for further exploration.

### 3.3 LINC00941 was an independent prognostic and early diagnostic factor for LUAD patients

**FIGURE 3:**
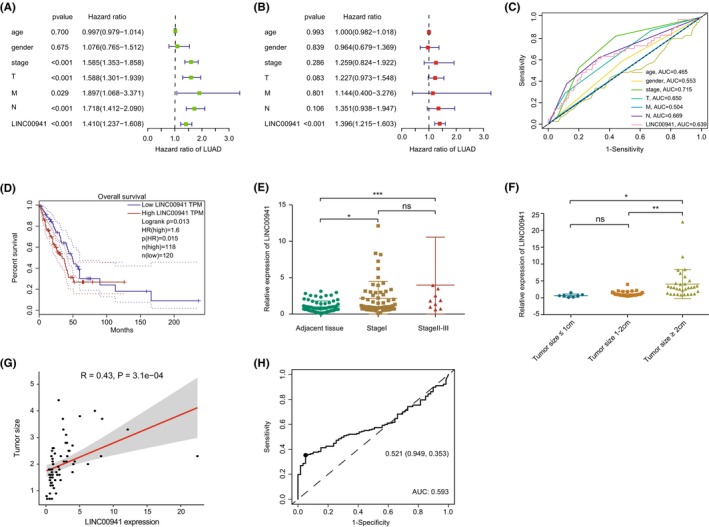
LINC00941 exhibited independent prognostic and early diagnostic value in patients with LUAD. (A, B) The results of univariate and multivariate Cox regression analyses. (C) The AUC values of LINC00941 and other clinical characteristics. (D) Kaplan–Meier curves demonstrated distinct prognostic outcomes among LUAD patients based on LINC00941 expression value. (E, F) The expression levels of LINC00941 in 67 pairs of clinical samples with different stages (adjacent, *n* = 67; stage I, *n* = 57; stage II–III, *n* = 10) and tumour sizes (≤1 cm, *n* = 7; 1–2 cm, *n* = 29; ≥2 cm, *n* = 31) were determined by RT‐qPCR. Data were analysed by one‐way ANOVA followed by the Tukey's post hoc test. (G) Pearson correlation analysis of LINC00941 expression with tumour size based on 67 pairs of LUAD clinical samples. (H) ROC curves were performed to assess the sensitivity and specificity of LINC00941 in distinguishing stage I LUAD tissues and normal tissues based on TCGA database. *p < 0.05, **p < 0.01, ***p < 0.001.

Further analysis revealed that the LINC00941 expression was an independent factor in predicting the prognosis of LUAD patients (Figure 3A,B). The AUC value for predicting patients' prognosis based on the expression value of LINC00941 was 0.639, which was approximately equal to that of TNM stage (Figure 3C). According to findings from GEPIA database,^36^ the elevated expression of LINC00941 was associated with an unfavourable prognosis (Figure 3D). Moreover, LINC00941 expression levels remained higher in tumour groups than that in normal groups as the sample size was expanded to 67 pairs (Figure 3E). It was also found that, in LUAD patients, the expression of LINC00941 showed a significant increase as the tumour stage progressed, indicating a positive relation with TNM stage (Figure 3E and Table 1), which was also supported by data from TCGA database (Figure S2A). Interestingly, based on the RT‐qPCR and chest CT data from 67 pairs of clinical samples, we found that the expression of LINC00941 in tumour tissues was increased as the tumour size increased (Figure 3F), which was consistent with the results obtained based on the TCGA‐LUAD dataset (Figure S2B). In addition, by analysing the LINC00941 expression level in the 67 pairs of LUAD samples, we found that the expression of LINC00941 was significantly positive correlated with tumour size (Figure 3G). This result indicated that the tissue expression level of LINC00941 could distinguish tumours less than 2 cm. In addition, the ROC curves were also used to further assess the performance of LINC00941 for LUAD detection, and the results showed that the specificity and sensitivity were respectively more than 90% and around 35% (Figure 3H and Figure S3), which is better than classical markers.^8^ These findings suggest that LINC00941 is a prognostic factor with early detection potential and is closely associated with tumour size.

### 3.4 LINC00941 promotes LUAD cell proliferation both in vitro and in vivo

**FIGURE 4:**
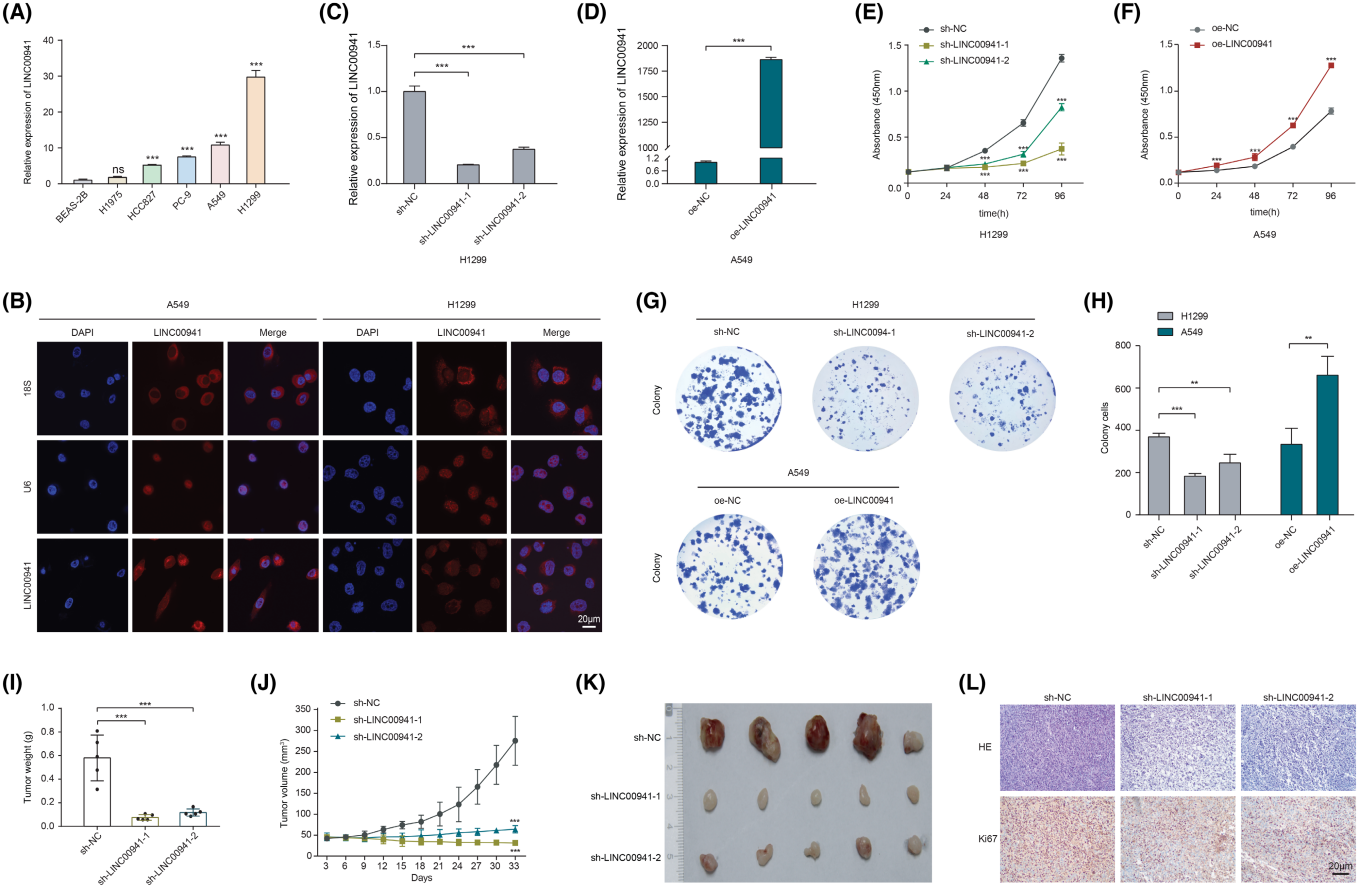
LINC00941 promoted cell proliferation in vitro and in vivo. (A) The expression levels of LINC00941 in LUAD cell lines and normal cell (BEAS‐2B). Data were analysed by one‐way ANOVA followed by the Dunnett's post hoc test and expressed as mean ± SD. (B) Subcellular localization of LINC00941 in LUAD cells by RNA FISH. (C, D) The knockdown and overexpression efficiency of sh‐LINC00941‐1/2 and oe‐LINC0094 were verified by RT‐qPCR. Data were analysed by two‐tailed Student's *t*‐test (oe‐NC vs oe‐LINC00941) or one‐way ANOVA followed by the Dunnett's post hoc test (sh‐NC vs. sh‐LINC00941‐1 or sh‐LINC00941‐2), and expressed as mean ± SD. (E, H) The determination of the cell viability with CCK8 assay (E, F) and colony forming efficiency with colony formation assay (G, H). Data were analysed by two‐tailed Student's *t*‐test (oe‐NC vs. oe‐LINC00941) or one‐way ANOVA followed by the Dunnett's post hoc test (sh‐NC vs. sh‐LINC00941‐1 or sh‐LINC00941‐2), and expressed as mean ± SD. (I–L) The weight (I), volume (J), image (K) and Ki67 IHC analysis (L) of subcutaneous xenograft tumours in sh‐NC, sh‐LINC00941‐1 and sh‐LINC00941‐2 groups (*n* = 5). Data were analysed by one‐way ANOVA followed by the Dunnett's post hoc test. **p < 0.01, ***p < 0.001.

The levels of LINC00941 in 5 LUAD cell lines were valuated (Figure 4A). According to its expression among the cell lines, A549 and H1299 were selected as the experimental cell model for subsequent analysis. The results of RNA FISH indicated that LINC00941 mainly localized in the cytoplasm in both cell lines (Figure 4B). To investigate the biological function of LINC00941, the expression of LINC00941 in H1299 was downregulated using shRNAs and was overexpressed in A549 using lentivirus carrying LINC00941 (Figure 4C,D). Knockdown of LINC00941 in H1299 cells significantly impaired the cells viability and attenuated the colony formation. In contrast, overexpression of LINC00941 in A549 cells markedly enhanced the viability and colony forming efficiency (Figure 4E–H).

Subsequently, we applied a subcutaneous tumorigenesis model to further assess the biological function of LINC00941 in vivo. Following stable transfection with sh‐LINC00941‐1, sh‐LINC00941‐2 or sh‐NC, H1299 cells were administered subcutaneously to nude mice. It was found that the tumour weight and volume were significantly lower in the two shRNA groups compared to the control group at 33 days of transplantation (Figure 4I–K). In addition, IHC staining was applied to detect Ki67 expression in tissues, an important indicator of tumour proliferation. A significant decrease in the number of Ki67‐positive cells was observed in the tumour tissues of the two shRNA groups (Figure 4L). These observations suggested that LINC00941 could enhance the tumour cell proliferation in vitro and accelerate tumour growth in vivo, and the depletion of it could cause opposite effects on LUAD development.

### 3.5 LINC00941 regulates tumour cell autophagy

**FIGURE 5:**
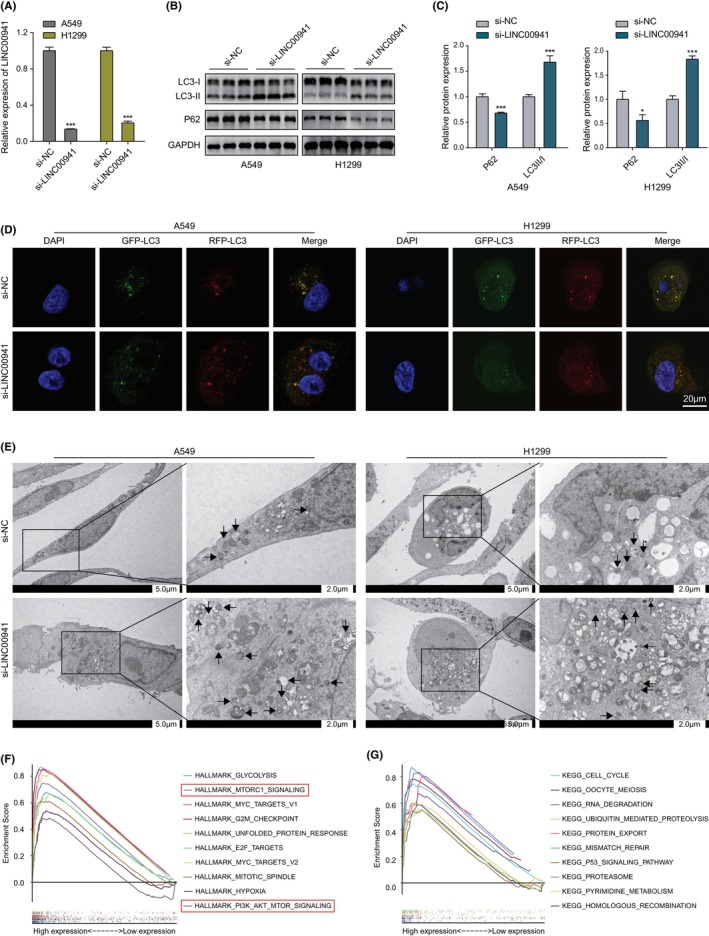
Knockdown of LINC00941 triggered autophagy. (A) The transfection efficiency of LINC00941 knockdown by siRNA was measured by RT‐qPCR. Data were analysed by two‐tailed Student's *t*‐test and expressed as mean ± SD. (B, C) The LC3 and P62 protein levels in LINC00941‐deficient cells were analysed by Western blotting analysis. Data were analysed by two‐tailed Student's *t*‐test and expressed as mean ± SD. (D) Images of mRFP‐GFP‐LC3 distribution in the knockdown groups and control groups were obtained with a confocal microscope. (E) The number of autophagosomes was measured by TEM. (F, G) The Hallmark and KEGG enriched pathways in the high‐risk cohort labelled by LINC00941 median expression. **p < 0.01, ***p < 0.001.

The siRNA was used to knockdown LINC00941 in both A549 and H1299, and its knockdown efficiency was about 86% in A549 and 80% in H1299 (Figure 5A), respectively.  The Western blotting analysis showed that the downregulation of LINC00941 significantly enhanced the transformation of LC3‐I to LC3‐II and the breakdown of P62 in A549 and H1299 cells (Figure 5B,C). Subsequently, the effect of LINC00941 on autophagy flux was assessed using the mRFP‐GFP‐LC3 double fluorescence lentivirus. The green fluorescence will decrease with the fusion of autophagosomes with lysosomes, leaving only the dot like or circular red fluorescence in acidic conditions. In contrast to the si‐NC‐treated cells, where only a slight increase in red puncta was observed, a dramatic increase was detected in the si‐LINC00941‐treated cells, indicating an enhanced autophagy flux in LINC00941 deficient cells (Figure 5D). Furthermore, TEM was performed to observe autophagosomes. A549 and H1299 cells treated with si‐LINC00941 showed more numbers of double‐membrane autophagosomes and autolysosome than that of the control group (Figure 5E). To further explore the mechanisms by which LINC00941 regulates proliferation and autophagy, we employed GSEA and identified pathways related to cells proliferation and autophagy, including the cell cycle, G2M checkpoint, MYC targets V1/V2, PI3K/AKT/mTOR, mTORC1, ubiquitin‐mediated proteolysis and P53 pathways, which were enriched in the LINC00941 high‐expression groups (Figure 5F,G). Based on the aforementioned observations, it can be inferred that downregulation of LINC00941 triggered autophagy in LUAD cells, which may be associated with the aforementioned pathway, particularly the PI3K/AKT/mTOR pathway.

## 4 DISCUSSION

In the present study, by performing bioinformatics analysis, we found that the tissue levels of LINC00941 demonstrated a sensitivity of approximately 35% for LUAD early diagnosis, exceeding that of the classical markers.^8^ Moreover, the expression of LINC00941 was found to vary with tumour sizes in LUAD patients, especially in small tumours (size <2 cm) that cannot be easily detected by low‐dose CT. Previous study has demonstrated that LINC00941 act as a pro‐transcription factor of oncogenes in diverse malignancies, such as hepatocellular carcinoma,^26^ oesophageal squamous cell carcinoma,^37^ oral squamous cell carcinoma,^38^ pancreatic cancers,^39^, ^40^ thyroid cancer^41^ and colorectal cancer.^42^ In these studies, it was clearly shown that the high expression of LINC00941 was associated with poor prognosis, and played a role in promoting proliferation, metastasis and angiogenesis. Consistent with these studies, our research revealed that LINC00941 promotes tumour proliferation in vitro and enhances tumour growth in vivo. Specifically, the deficiency of LINC00941 in tumour cells significantly inhibited cell proliferation, while its overexpression had the opposite effect. Therefore, LINC00941 emerges as a promising biomarker for early detection and a target for therapeutic intervention in LUAD.

It is now widely accepted that autophagy may contributed to the survival of established cancer.^43^ However, several studies have indicated that autophagy supports the established cancer death.^44^, ^45^ The seemingly contradictory observations suggest that the exact effects of autophagy in established cancer may depend on different contexts, especially the inflammatory response in the tumour microenvironment (TME). P62 is known as not only an indicator of autophagy flux but also a regulator of inflammation response.^46^ Specifically, the accumulation of P62 leads to an enhanced inflammatory response, which promotes tumour progression.^47^, ^48^ Correspondingly, inhibition of P62 accumulation has the opposite effect. Moreover, the inflammatory responses participate in every process of tumour progression, including proliferation, angiogenesis and invasion.^49^, ^50^ LncRNA MALAT1 is one example in which silencing MALAT1 in gastric cancer cells triggered autophagy via mTOR inhibition, thereby suppressing cell proliferation.^44^ Specifically, the inhibition of P62 accumulation based on autophagy activation resulted in IL‐6 secretion impairment associated with NF‐κB signalling in the TME.^44^ In this study, we found that, for the first time, the downregulation of LINC00941 significantly enhanced the breakdown of P62 and the transformation of LC3‐I to LC3‐II, resulting in an elevated level of autophagy flux in A549 and H1299 cells. LINC00941 deficiency significantly inhibited the accumulation of P62 based on autophagy enhancement. For proliferation regulation, the downregulation of LINC00941 inhibited cell proliferation. The overexpression of LINC00941 caused the opposite effect. In this context, the enhancement of autophagy induced by LINC00941 deficiency may result in tumour suppressive effects.

Furthermore, several signalling pathways involving in the regulation of autophagy, such as ubiquitin‐mediated proteolysis, P53 pathway, mTORC1 and PI3K/AKT/mTOR pathway, were found to be enriched in the high‐expression groups of LINC00941.^51^, ^52^, ^53^ Among them, the PI3K/AKT/mTOR pathway is extensively studied. The activation of mTOR sustains the proliferative anabolism via blocking autophagosome formation mediated by ULK1, ATG14 and ATG13, as well as the fusion between autophagosome and lysosome.^54^, ^55^ In turn, the accumulation of P62 could be responsible for mTOR activation through its interaction with raptor that is one of the positive regulatory subunits of mTORC1, concurrently inducing inflammatory response and cell proliferation.^46^, ^56^ A crosstalk would be formed between mTOR, autophagy and inflammation. Here, we found a significant enrichment of the PI3K/AKT/mTOR pathway in the cohort with high expression of LINC00941. Considering the effects of LINC00941 on cell proliferation and autophagy, it is postulated that LINC00941 may potentially impact cellular autophagy and proliferation by the PI3K/AKT/mTOR pathway. However, additional experiments are necessary to validate and support our hypothesis. Pathway‐based inhibitors or activators are needed to evaluate the rescue effect of the PI3K/AKT/mTOR pathway on LINC00941‐mediated biological functions. Rescue experiments were done to confirm if LINC00941 can regulate autophagy and proliferation via the PI3K/AKT/mTOR pathway. RNA FISH indicated that LINC00941 mainly localized in the cytoplasm in LUAD cells. Previous studies have demonstrated that LINC00941 exerted biological functions through post‐transcriptional regulatory mechanisms, such as the RNA‐protein interaction or the competing endogenous RNA. Hence, further experiments may be around to explore the mechanistic link between LINC00941 and PI3K/AKT/mTOR pathway.

In summary, our findings underscore the significance of LINC00941 as an adjunct to low‐dose CT in the early diagnosis and therapeutic intervention of LUAD. Particularly, the cancer‐promoting effects of LINC00941 in various tumours provided evidence of the generalizability significance of LINC00941‐targeting therapy. However, RNA‐based therapeutics face challenges in terms of specificity, delivery and tolerability in clinical settings.^57^ Targeting LINC00941 as a therapy may present a promising approach, but it also brings concerns about off‐target effects and immune‐related adverse effects.

## AUTHOR CONTRIBUTIONS

**Qin Yang:** Conceptualization (equal); methodology (equal); writing – original draft (equal). **Xi Yong:** Formal analysis (equal); methodology (equal). **Xiaoli Chen:** Methodology (equal); resources (equal). **Rong Huang:** Formal analysis (equal); methodology (equal). **Xiaolin Wang:** Methodology (equal); resources (equal). **Zhengmin Xu:** Conceptualization (equal); writing – review and editing (equal). **Wei Chen:** Conceptualization (equal); funding acquisition (equal); writing – review and editing (equal).

## FUNDING INFORMATION

This work was supported by Natural Science Foundation of Sichuan (No. 2023NSFSC0683).

## CONFLICT OF INTEREST STATEMENT

None declared.

## Supporting information


Appendix S1.

## Data Availability

Data available on request from corresponding authors.
