# Supplementary material for: LINC00941 is a diagnostic biomarker for lung adenocarcinoma and promotes tumorigenesis through cell autophagy
Source: J Cell Mol Med. 2024 Oct 11;28(19):e70076. doi: 10.1111/jcmm.70076 (PMC11467743; doi:10.1111/jcmm.70076)
Supplement: Supplementary file 1 — Appendix S1. [file JCMM-28-e70076-s001.zip › Supplementary Materials.docx]

**Supplementary Materials**

**LINC00941 inhibits cell autophagy via activating PI3K/AKT/mTOR signaling pathway to promote the progress of lung adenocarcinoma**

**Qin Yang^1^****^,2^, Xi Yong^3^, Xiaoli Chen^4^, Rong Huang^5^, Xiaolin Wang^4^, Zhengmin Xu^5,6^*, Wei Chen^1,2^***

^1^School of Basic Medical Sciences, Chengdu University of Traditional Chinese Medicine, Chengdu 611137, China

^2^Innovative Institute of Chinese Medicine and Pharmacy, Chengdu University of Traditional Chinese Medicine, Chengdu 611137, China

^3^Department of Vascular Surgery, Affiliated Hospital of North Sichuan Medical College, Nanchong 637000, China

^4^Department of Pathology, Basic Medicine and Forensic Medicine College, North Sichuan Medical College, Nanchong 637000, China

^5^School of Pharmacy, Institute of Materia Medical, North Sichuan Medical college, Nanchong 637000, China

^6^Traditional Chinese Medicine for Prevention and Treatment of Musculoskeletal Diseases Key Laboratory of Nanchong City, Nanchong 637000, China

*Correspondence should be addressed to Zhengmin Xu: [xuzhengmin@nsmc.edu.cn](mailto:xuzhengmin@nsmc.edu.cn); Wei Chen: [greatchen@ncst.edu.cn](mailto:greatchen@ncst.edu.cn)

**1 Supplementary Table**

Table S1. The RT-qPCR primer sequences involved in this study.

| Gene | Sequence | |
| --- | --- | --- |
| β-actin | Forward | 5′-GCACAGAGCCTCGCCTT-3′ |
|  | Reverse | 5′-GTTGTCGACGACGAGCG-3′ |
| LINC00941 | Forward | 5′-CAGAGTCCCTCTGAGACCAA-3′ |
|  | Reverse | 5′-ACTTGGGAGACTGAGATGGA-3′ |
| HIF1A-AS1 | Forward | 5′-CACACGCGGAGAAGAGAAG-3′ |
|  | Reverse | 5′-GGTGCGTAAAGTACCGAAGAA-3′ |
| TMPO-AS1 | Forward | 5′-CTTTTGTGCGCCGTTTCCT-3′ |
|  | Reverse | 5′-CCCAGAGACGAAAGCTGCTT -3′ |
| LINC01150 | Forward | 5′-CGGAAGCAAAGCACAGAAAC-3′ |
|  | Reverse | 5′-AGCTATGATGGCACCACTG-3′ |

Table S2. To mining lncRNAs associated with autophagy, a total of 77 lncRNAs were acquired through univariate Cox analysis based on TCGA-LUAD dataset. Original data are accessible with the “Table_S2.”

**2 Supplementary Figures**


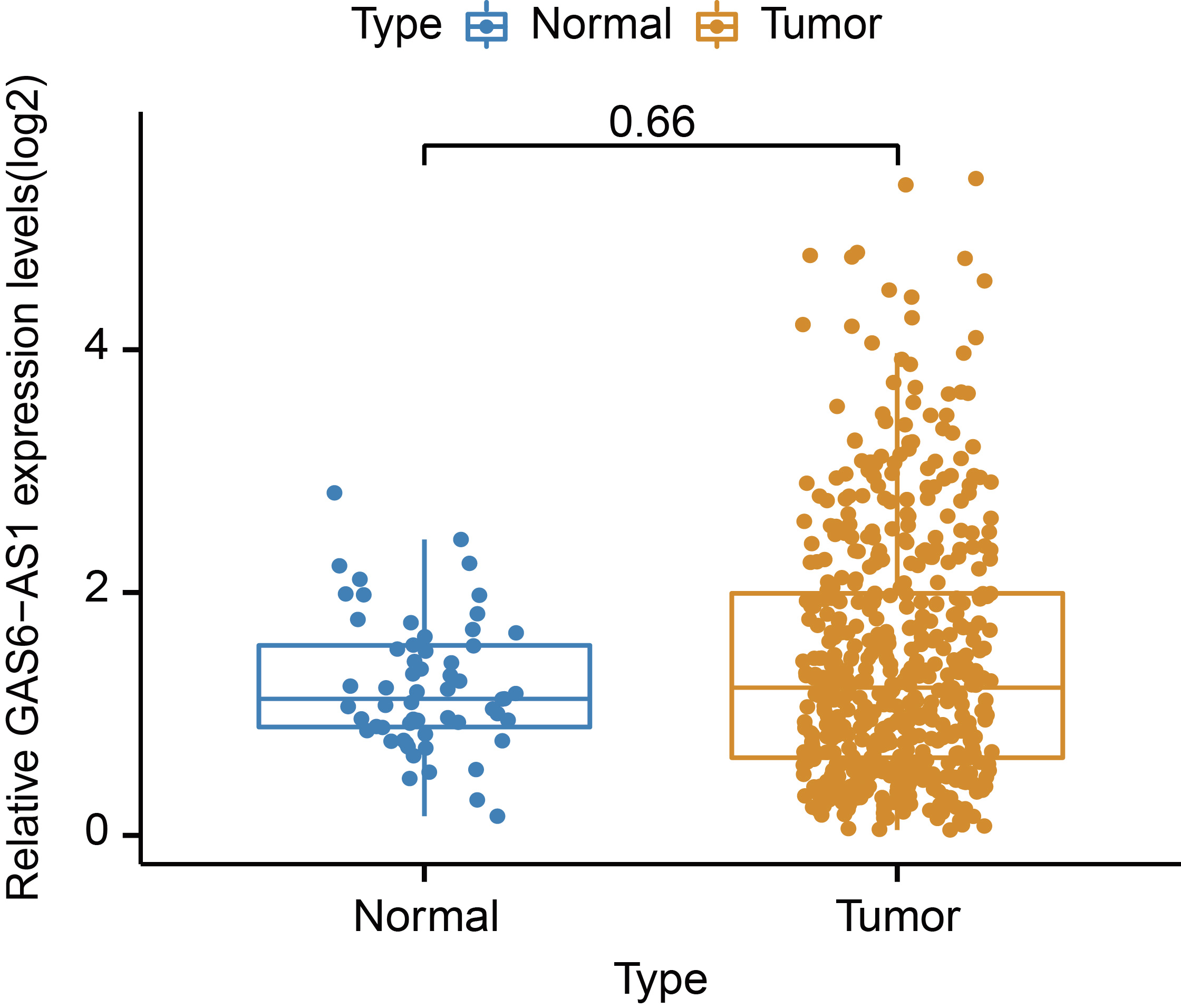


**Figure S1** The expression level of GAS6-AS1 based on TCGA database.


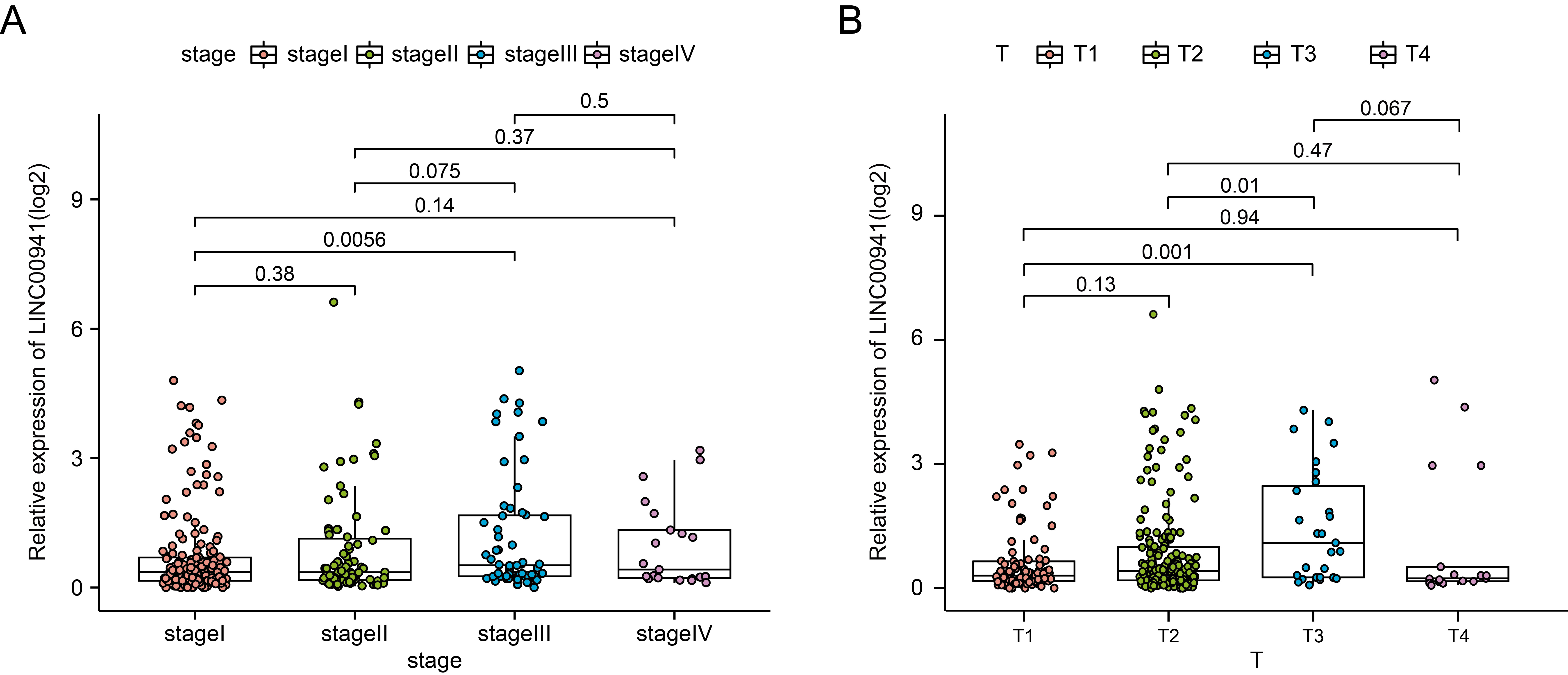


**Figure S2** Correlation of LINC00941 expression with (A) TNM stage (stage I, n = 167; stage II, n = 79; stage III, n = 59; stage IV, n = 21) and (B) tumor size (T1, n = 98; T2, n = 184; T3, n = 27; T4, n = 17) based on TCGA-LUAD dataset.


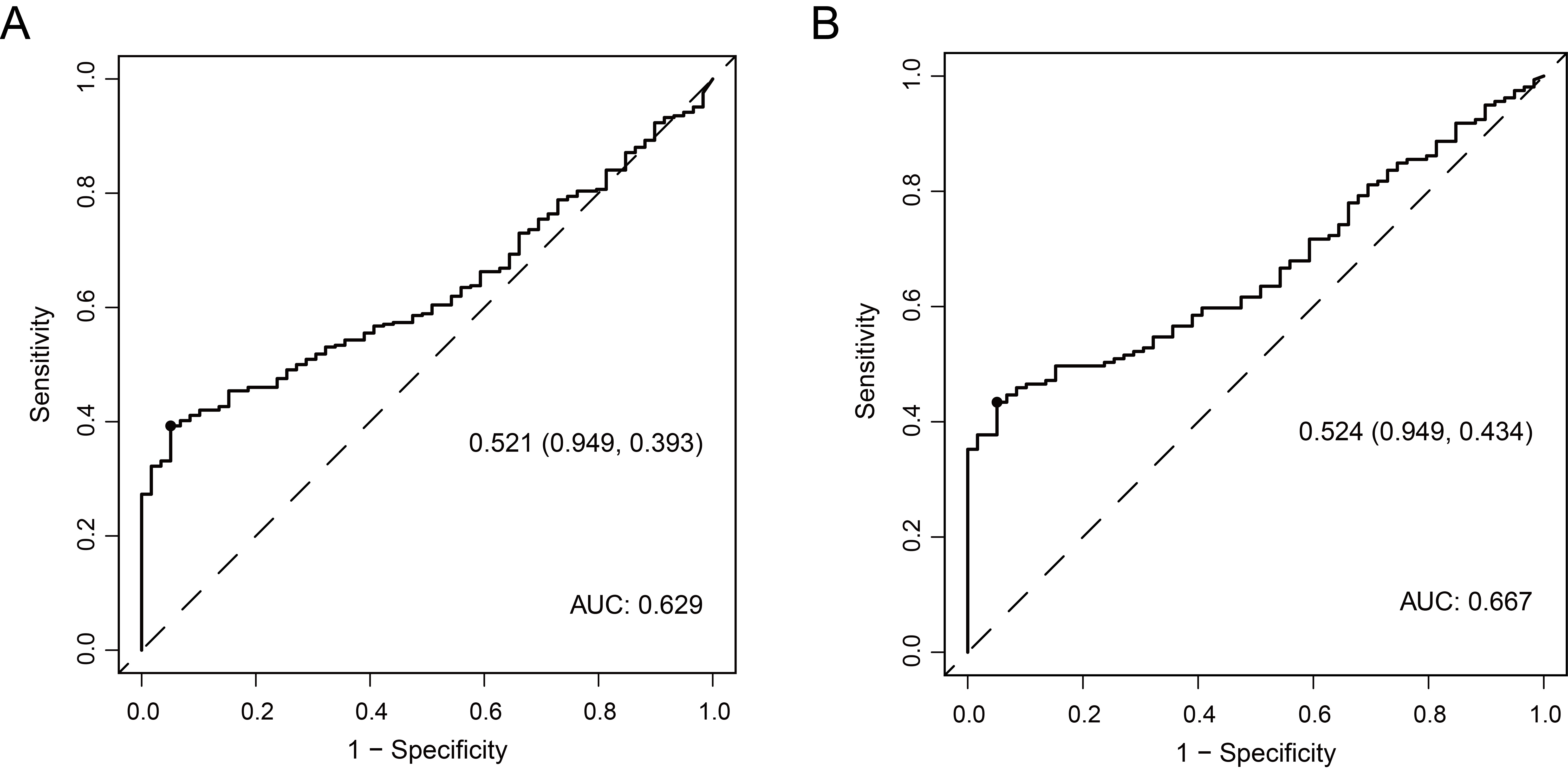


**Figure S3** ROC analysis of LINC00941 levels for distinguishing (A) LUAD tissues and normal tissues, and (B) stage II-IV LUAD tissues and normal tissues based on TCGA database.
